# Development and validation of entrustable professional activities in pediatric surgery for pediatricians

**DOI:** 10.1016/j.jped.2025.101444

**Published:** 2025-09-30

**Authors:** Rodrigo Pinheiro de Abreu Miranda, Edna Regina Silva Pereira, Leopoldo Luiz dos Santos-Neto

**Affiliations:** a Associação Brasileira de Cirurgia Pediátrica (CIPE), Brazil; b Hospital da Criança de Brasília José Alencar (HCB), Programa de Residência em Cirurgia Pediátrica, Brasília, DF, Brazil; c Universidade Federal de Goiás (UFG), Departamento de Medicina Interna, Goiânia, GO, Brazil; d Universidade de Brasília (UnB), Faculdade de Medicina, Programa de Pós-Graduação em Ciências Médicas, Brasília, DF, Brazil

**Keywords:** Medical education, Internship and residency, Pediatric assistants, Competency-based education, Professional competence

## Abstract

**Objective:**

To develop and validate entrustable professional activities (EPAs) for the training of pediatric residents on topics that interface with pediatric surgical areas in the Brazilian context.

**Methods:**

The study was conducted in two phases. In the first phase, experts were oriented and contextualized, and they were responsible for developing the initial list of EPAs. In the second phase, the Delphi technique was applied in three rounds: the first for consensus, the second for selection according to relevance and agreement, and the third for validation and detailing of the EPAs.

**Results:**

In the first phase of the study, 9 experts listed 88 EPAs, which were applied in the Delphi method. In the first round of Delphi, the consensus of these experts defined 31 EPAs, with CVI ≥ 0.80, and ICC of 0.893 (95 % CI 0.823–0.945). In the second round, 25 coordinators of Medical Residency Programs selected 17 EPAs by agreement and relevance (CVI ≥ 0.80, and ICC of 0.851–95 % CI 0.753 to 0.924). In the third round, 50 preceptors from all over Brazil validated 14 EPAs with CVI ≥ 0.965 and ICC 0.866 (95 % CI 0.804–0.915), which were organized and detailed into 7 final EPAs.

**Conclusion:**

Seven pediatric surgery EPAs were developed, consensualized, selected, and validated by experts for the work of pediatricians in Brazil through the Delphi method. The great participation and interest of medical residency preceptors with a wide geographical coverage in Brazil were strong points of this study, and these EPAs can be applied, reviewed, and updated.

## Introduction

The 21st-century medical resident is immersed in a socio-cultural reality vastly different from that of the early 20th century when Osler and Halsted founded residency programs. In this context, the competency-based medical education (CBME) model emerged in the early 1990s as a framework for undergraduate and postgraduate medical education [1,2]. This model suggests that professionals should develop specific and comprehensive competencies, including knowledge, skills, and attitudes, that are durable, teachable, measurable, related to professional activities, and connected to other individual competencies desirable for excellence in their training [1, 2, 3]. CBME defines objectives to be achieved at the end of training and has been widely adopted in medical schools and residency programs worldwide [2,3]. However, CBME has been criticized for being overly theoretical, difficult to verify in practice, and having limited applicability in the individual assessment of trainees [3].

To address these challenges and provide a more practical teaching tool, Dutch physician and educator Theodorus Jan (Olle) Ten Cate introduced the concept of Entrustable Professional Activities (EPAs) in 2005 [4]. EPAs are essential tasks that professionals must be able to perform independently by the end of training, serving as a bridge between competencies and clinical practice [4,5]. When implemented effectively, EPAs allow students, instructors, and even patients to have a clear understanding of the physician’s current scope of practice [5,6].

According to Ten Cate, EPAs do not replace competencies but rather translate them into clinical practice, allowing the training physician progress toward specialization, to independently care for the patient [5,7]. When this model is conducted clearly, both students and instructors, as well as patients themselves, can better understand what the physician is or is not qualified to do [8–9].

Pediatrics is one of the many medical fields where most professionals are trained through residency programs, which are facing significant challenges in ensuring high-quality training [10,11]. In this scenario, competency matrices with their respective EPAs have been developed and validated internationally, both for general pediatrics and pediatric subspecialty residency programs [11, 12, 13, 14]. However, few well-designed studies have been conducted on the subject, as the concepts of EPAs are still, in their essence, poorly understood [7,14].

In Brazil, interest in EPAs has been growing among medical educators, especially at the undergraduate level, and is an increasingly present topic in forums, courses, congresses, and publications [15, 16]. However, its application in the training of resident physicians is still in its early stages [16]. In Pediatrics, the authors can praise the pioneering work of Costa and collaborators, who recently validated EPAs for pediatric intensivists in Brazil [17].

In this context, the competency matrix for pediatric residency programs, published by the National Medical Residency Commission in 2016 and implemented in 2019 [18], determines that the resident physician must acquire knowledge, skills, and attitudes necessary for managing the most prevalent pediatric surgical diseases [18]. This directive underscores the need to enhance resident training through the development of a curricular model that incorporates EPAs related to pediatric surgery, tailored to general pediatricians – a gap that has yet to be addressed.

Therefore, this unprecedented study in Brazil proposes, through the Delphi method, to develop and validate EPAs for the training of pediatric residents in topics that interface with pediatric surgical areas, while still aligned with the Brazilian competency matrix. This pioneering work seeks to fill a gap in national medical education, contributing to the improvement of the quality of teaching and care in Pediatrics.

## Methods

### Design, setting, and participants

This study was conducted in two phases to establish Pediatric Surgery EPAs for general pediatric residency programs in Brazil. To assemble the study's expert panel, the primary criterion was experience in Pediatric Surgery and in the training of general pediatric residents, aiming for the broadest possible geographic representation in Brazil. The first phase involved orientation and contextualization of the work group, followed by the development of a list of all professional activities that interface with Pediatric Surgery that a General Pediatrician should perform in their career. In the second phase, the Delphi technique was applied in three rounds: the first for consensus, the second for selection based on relevance and agreement, and the third for validation and detailing of the EPAs.

This study was submitted to the Ethics Committee and approved under the CAAE number 55420822.0.0000.0144.

### Data collection and analysis

During the second phase, to apply the Delphi method with three rounds among experts for consensus, selection, and validation of Pediatric Surgery EPAs for general pediatric residents in Brazil, structured questionnaires were used to collect and record data through the REDCap® platform. The questionnaire was distributed to experts by WhatsApp®, a free and secure, cross-platform instant messaging app. Data analysis was performed using MS Excel (Microsoft Office Professional Plus, 2013) and IBM SPSS (Statistical Package for the Social Sciences v. 23.0, 2015).

### First phase of the study: expert panel

For this phase, professionals in the position of supervisors/coordinators of Pediatric Surgery Residency Programs, all with experience in training general pediatric residents, and specialist members of the CIPE (Brazilian Pediatric Surgery Association) were contacted and invited via telephone. Initially, orientations were provided on the EPA curricular model and the Delphi method, including the forwarding of material on the subjects for a better understanding of the study.

Subsequently, the experts were individually instructed to evaluate and propose potential Pediatric Surgery EPAs for training general pediatric residents. The completion time for this phase was six weeks, during which clarifications about the subject and working method were provided by the study coordinators.

At the end of the first phase, duplicate or highly similar entries were excluded, to ensure that the proposed EPAs met the established criteria: essential activities are understandable and assessable.

### Second phase: delphi method

#### Round 1 – consensus

In this round, a questionnaire was developed to facilitate content consensus analysis. The Entrustable Professional Activities (EPAs) identified by experts in the first phase were compiled and sent to all experts for peer review and evaluation, covering all 88 EPAs. This round lasted three weeks, and experts assessed the importance of each EPA using a five-point rating scale: “not important,” “mildly important,” “important,” “very important,” and “indispensable.”

#### Round 2: selection by relevance and agreement

In this stage, three weeks after the previous round, the experts were instructed to determine the representativeness and relevance of the content of each EPA. The scale used was the same as in round 1, ranging from 1 to 5, from not important to indispensable. A four-week deadline was set for the completion of this phase. After completion, the results of round 2 were compared with those of round 1 to further assess relevance and agreement.

The statistical analysis of the first two rounds of the Delphi method was divided into descriptive analysis, content validity, and reliability analysis. Qualitative variables were described using frequencies and percentages. The Content Validity Index (CVI) in this study was calculated by the mean value. The answers “very important” and “indispensable” were considered adequate, and the value 1 was assigned to them in each situation. The CVI corresponds to the average of the item values. The intraclass correlation coefficient (ICC) evaluates the agreement between more than two data sets or more than two raters. Currently, it is also a widely used value to determine the validity of an instrument through the agreement between judges (experts). An ICC close to 1 indicates high agreement among the values of the same group, and a low ICC close to zero expresses low agreement among the values. To calculate the ICC, the Likert scale initially used at five levels (0 to 4) was considered.

#### Round 3: validation and detailing

This stage of the Delphi study lasted two months and aimed to validate the Pediatric Surgery EPAs that are fundamental to the training of general pediatric residents. For this stage of the study, Brazilian Pediatric Surgery professionals who act as preceptors for resident physicians in both surgical and clinical areas of child care were invited.

The ideal sample size was determined based on the ICC of the previous phase. For a minimum ICC of 0.80 (p) and an acceptable error of 0.20 (wp) with a 95 % confidence interval, using the formula: 1+ [8(1,96)2(1 – p)2(1 + *p*)2/(2wp)2], the authors have *n* = 50 preceptors of Pediatric Residency programs. To assess the level of agreement and validation, a five-item rating scale was developed, ranging from strongly disagree to strongly agree. The questionnaire included demographic data, level of specialization, professional experience, and experience as a residency program preceptor.

The statistical analysis of this phase of the study was also divided into descriptive analysis, content validity, and reliability analysis. For the content validity of round 3, the answers “agree” and “strongly agree” were considered adequate, and the value 1 was assigned to them in each situation, where the CVI corresponds to the average of the item values. To calculate the ICC of this stage of the Delphi study, the Likert scale used was evaluated at five levels (1 to 5) of agreement.

Following the completion of the validation process, the Pediatric Surgery EPAs were organized, grouped, and detailed to align with the scope of general pediatric practice. This structuring enables comparative analysis and supports its implementation as a curricular model, preserving both the breadth and specificity required for effective pediatric care.

## Results

**Figure 1:**
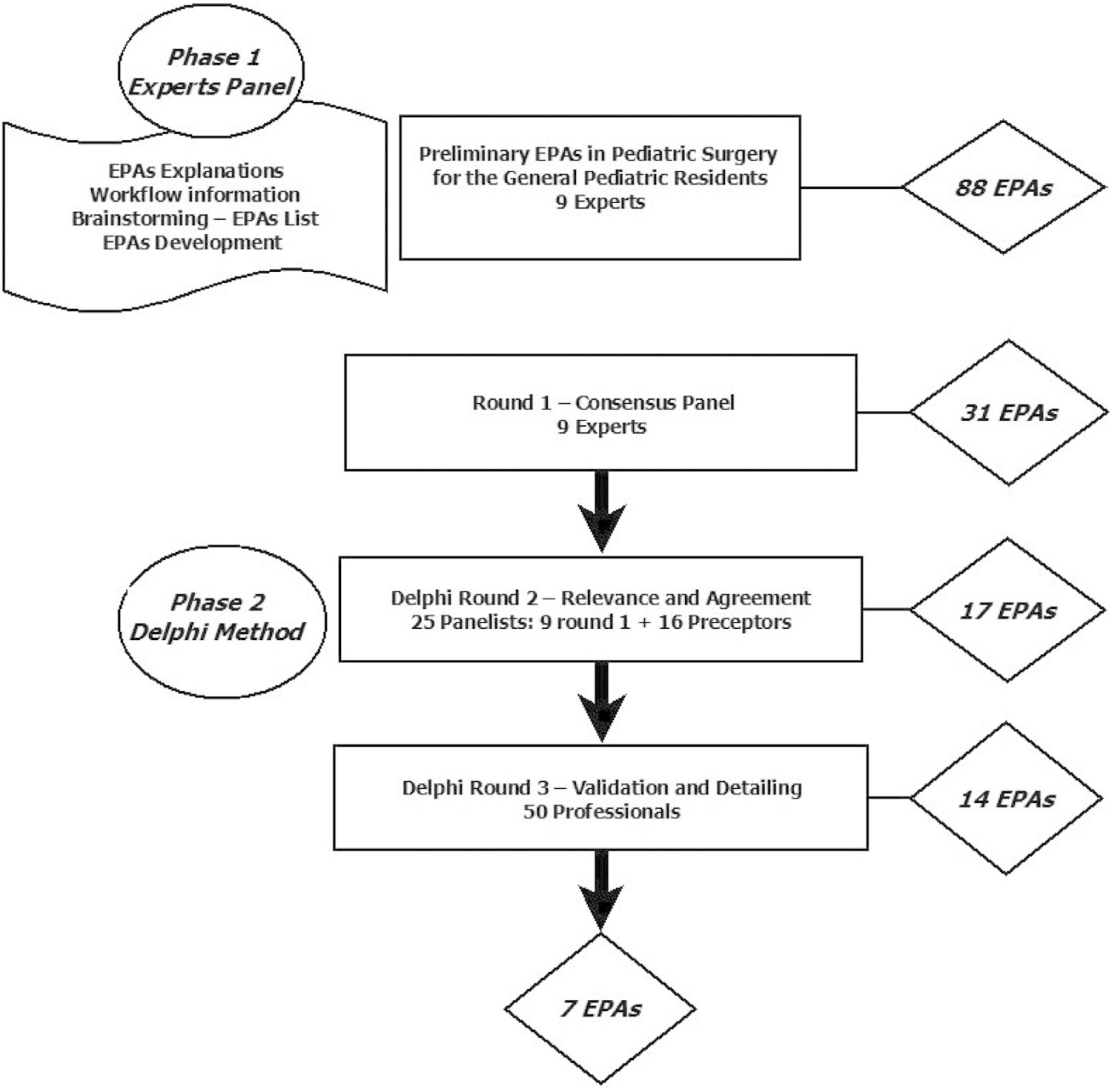
Development and Validation of EPAs for Pediatric Residents in Pediatric Surgical Conditions.

For the first phase of the study, 12 residency program supervisors/coordinators with specialist titles were invited from across Brazil’s five geographic regions to ensure coverage of the diverse realities in Brazilian pediatric training. Of these, nine professionals agreed to participate, three from the Southeast region, two from the Northeast, two from the North, one from the Midwest, and one from the South region. These specialists initially developed a total of 88 Pediatric Surgery EPAs for the training of pediatric residents in Brazil (Figure 1).

In the first round of the Delphi method (consensus stage) of the second phase, the questionnaire was applied through the REDCap® platform to the initial nine specialists regarding the 88 activities listed in the first phase of the study. The responses were evaluated and analyzed, obtaining consensus on 31 EPAs, with CVI ≥ 0.80, for the categories “very important” and “indispensable”, and ICC of 0.893 (95 % CI 0.823 – 0.945) - Figure 1.

For the second round of the Delphi method (to define relevance and agreement), invitations were sent to all supervisors/coordinators of 54 Brazilian Pediatric Surgery residency programs. At this stage, 25 Pediatric Surgery preceptors participate in the training of general pediatric residents (9 specialists from phase 1 and 16 new preceptors), with a wide geographical distribution throughout the country, as shown in Table 1. From the 31 EPAs of the first round of the Delphi method, 17 EPAs were selected, for the answers “very important” and “indispensable” with CVI ≥ 0.80, and ICC of 0.851 (95 % CI 0.753 – 0.924) - Figure 1.

**Table 1:** Descriptive analysis of the professional data of supervisors and coordinators of Pediatric Surgery Residency Programs who participated in the Delphi method – rounds 1 and 2.

Characteristic	State/ Region	n	%
In which State do you work?	North Region	2	8.0
	Acre (AC)	1	
	Tocantins (TO)	1	
	Northeast Region	5	20.0
	Bahia (BA)	1	
	Ceará (CE)	1	
	Paraíba (PB)	1	
	Pernambuco (PE)	1	
	Piauí (PI)	1	
	Midwest Region	1	4.0
	Distrito Federal (DF)	1	
	Southeast Region	13	52.0
	Minas Gerais (MG)	2	
	Rio de Janeiro (RJ)	3	
	São Paulo (SP)	8	
	South Region	4	16.0
	Paraná (PR)	2	
	Santa Catarina (SC)	2	
Role in Preceptorship / Medical Residency	Supervisor/Coordinator	19	76.0
	Preceptor	5	20.0
	Volunteer Preceptor	1	4.0
Total		25	100.0

The third round of the Delphi method was for the validation of the EPAs selected so far, and in this part of the work, from a total universe of approximately 450 preceptor physicians, 56 professionals (12.4 %) in Pediatric Surgery returned, of which two did not sign the informed consent form, three were duplicates, and one was incompletely filled out. Thus, 50 responses were considered valid and included in this study (Table 2).

**Table 2:** Descriptive analysis of professional data from preceptors, supervisors and coordinators of Pediatric Surgery Medical Residency programs who participated in the Delphi - Round 3.

Characteristic	State	N	%
In which state do you work?	Acre (AC)	1	2.0
	Tocantins (TO)	1	2.0
	Piauí (PI)	1	2.0
	Ceará (CE)	1	2.0
	Paraíba (PB)	1	2.0
	Pernambuco (PE)	3	6.0
	Bahia (BA)	4	8.0
	Goiás (GO)	1	2.0
	Distrito Federal (DF)	4	8.0
	Minas Gerais (MG)	3	6.0
	Espírito Santo (ES)	1	2.0
	Rio de Janeiro (RJ)	8	16.0
	São Paulo (SP)	12	24.0
	Paraná (PR)	6	12.0
	Santa Catarina (SC)	3	6.0
Working Region	North	2	4.0
	Northeast	10	20.0
	Midwest	5	10.0
	Southeast	24	48.0
	South	9	18.0
What is your activity in the medical residency preceptorship?	Attending Physician	1	2.0
	Preceptor	20	40.0
	Supervisor/ Coordinator	29	58.0
What is your level of teaching specialization in pediatric surgery?	Medical Residency	21	42.0
	Postgraduate in Preceptorship	2	4.0
	Master's Degree	13	26.0
	PhD’s Degree	12	24.0
	Other	2	4.0
Total		50	100.0

The questionnaire in this round assessed the agreement and validation of the 17 EPAs identified in the previous two Delphi rounds. Considering responses of “agree” and “strongly agree” as adequate, the calculated CVI was 0.965. However, three of the 17 previously selected EPAs had a CVI below 0.96, despite having been considered relevant in the earlier phase (Figure 1). The ICC for this round was 0.866 (95 % CI: 0.804–0.915).

In the detailing phase, to enhance clarity and applicability, the 14 EPAs validated in the third Delphi round were organized and condensed into seven broader EPAs aligned with general pediatric practice. For example, two EPAs related to outpatient surgical assessment were merged into a single EPA focused on the clinical evaluation and referral of children with suspected surgical conditions. Likewise, EPAs on inguinal and genital assessment were integrated into one comprehensive EPA. Neonatal surgical diagnoses, emergency care scenarios, and postoperative management were also grouped according to clinical context and overlapping required competencies. This reorganization preserved the essential content of each original EPA while improving coherence and feasibility for implementation. The final seven EPAs are presented in Table 3.

**Table 3:** Content Validity Index (CVI) and Detailed Analysis – Round 3 of the Delphi Method.

Entrustable Professional Activities (EPA)	I-CVI
Pediatric care of patients with suspected surgical conditions: obtaining a medical history, performing an adequate physical examination, establishing diagnostic hypotheses and differential diagnosis, and classifying the surgical disease for appropriate referral for surgical evaluation.	1.0
Evaluation of the child's inguinal and genital region: providing guidance on hygiene and local care, and diagnosing possible alterations in these areas requiring surgical intervention, including possible complications and establishing initial clinical management.	1.0
Pediatric care of children with surgical conditions in an urgent and emergency setting: resulting from trauma or otherwise, involves performing the diagnostic process and initial care.	1.0
Evaluation and general care of surgical wounds and stomas: performing initial management of complications, including reinsertion of devices in case of loss (cannulas, catheters, and collection bags).	0.98
Diagnostic evaluation and initial management of newborns in the delivery room and nursery with high-prevalence pediatric surgical conditions: esophageal atresia, congenital diaphragmatic hernia, anorectal anomalies, gastroschisis and omphalocele, necrotizing enterocolitis, and posterior urethral valve.	0.98
Performing clinical care of patients with surgical conditions under hospitalization: performing admission and preoperative preparation, as well as postoperative clinical evaluation.	0.96
Care of a child with pneumonia complicated by pleural disease: diagnostic evaluation, classification, and management of pneumonia complicated by pleural effusion and/or pneumothorax, including thoracentesis and chest tube management.	0.96
ICC: 0.866 (95 % CI 0.804 – 0.915)	
I-CVI: Individual Item Content Validity Index. ICC: Intraclass Correlation Coefficient.	

## Discussion

The present study, conducted with professionals in Pediatric Surgery, preceptors of Medical Residency Programs (MRPs), from all five geographic regions of Brazil, allowed for the validation of seven EPAs in surgical areas for the practice of Pediatricians in the country. As this work addresses an innovative topic in medical education, the development, validation, and implementation of a curriculum based on EPAs are challenging [3,19, 20].

In 2021, the American Board of Pediatrics (ABP) published on its website guidelines for the development of EPAs for the training of general pediatricians in a North American setting [21]. Even with the endorsement of one of the largest pediatric societies in the world, these guidelines have been criticized, mainly regarding their practical implementation and the ability to assess trainees [22].

One of the major obstacles observed in the development and validation of a curricular model with EPAs is the establishment of clear, measurable, and universally accepted criteria [3,6,7,20]. These competencies, when coupled with the demands of modern society´s health needs, which are very dynamic, and the local realities that influence these needs, [14,22] require excellent training of professionals in the medical area [1,3,5]. Thus, regarding the curricular model with EPAs, different institutions and regions may have varied expectations and particularities, which can complicate the development of a standardized curriculum [14,22].

To minimize these difficulties and achieve the goal of developing, reaching consensus, on selecting, and validating EPAs, the Delphi method has been adopted as one of the most used approaches to date [23, 24, 25]. The Delphi technique is considered effective for determining expert consensus, especially where there is little or no empirical evidence on a given subject [26,27]. In addition, its application allows for the balancing of divergent aspects and the solution of vague or unanswered questions about a specific area of knowledge [27,28].

In phase 1 of the study, prior to the application of the Delphi method, it was essential to have experienced professionals in the training of medical residents [17,23,25]. Therefore, the authors performed an active selection of specialists with experience not only in Pediatric Surgery but also in general pediatrics training, an aspect considered positive by the authors of this study. All the specialists who participated in this phase held supervisory/coordination positions in MRPs and have a specialist title from CIPE, in addition to participating in the training of medical residents in Pediatric Surgery and/or General Pediatrics. This selection facilitated the compilation of an initial list of professional activities for the pediatrician with an interface in Pediatric Surgery that more closely reflects the Brazilian reality.

In a country of continental dimensions, with diverse realities, the application of the Delphi method for the second phase of the study was considered appropriate to establish a more significant and comprehensive assessment [23,26]. A very relevant aspect of the present study is the geographical representativeness of the specialists who answered the questionnaires in the three rounds of the Delphi, especially in the third. Studies by Jesus et al. and Bistorff-Silva et al. (2023) on the distribution of pediatric surgeons in Brazil have shown a concentration in the Southeast and South regions, followed by the Northeast, Midwest, and North, [29,30] a pattern similar to that observed in the present data.

Another highlight during the application of the method was the participation of a significant number of qualified professionals with proven experience in resident training. According to 2023 data, there are 1414 pediatric surgeons in Brazil, [30] of which more than half are involved in teaching, including preceptorship in medical residency [29]. Fifty questionnaires were answered appropriately in the validation phase of the study, respecting the minimum determined by sample size calculation. These results demonstrate a significant interest of the specialists in the topic of EPAs in the training of medical residents, and this participation is expressive when compared to other similar studies [23,25].

However, the authors emphasize that there are limitations that should be pointed out. Studies using the Delphi method are inherently subject to criticism, particularly if not well designed and controlled [26,28]. In addition, they may present with response bias (participants more interested in the topic may be more likely to respond), where the influence of dissident dominant opinions can influence the real training environment [26]. To minimize these issues, the authors made an effort to organize the conduct of the study according to previously established criteria.

And, despite the significant involvement of professionals in the teaching of Pediatric Surgery from all over Brazil, the little experience with still very innovative topics (EPAs and Delphi methodology) can be considered another limitation of the results of the present study [7,28]. A possible reflection of this inexperience of the participants was the fact that 3 of the EPAs considered relevant by the initial panel of professionals and specialists in the first two rounds of the Delphi method were not validated by the national group of preceptors. A comparable study involving medical education experts familiar with EPAs, or pediatric specialists, could be undertaken to verify the relevance of the activities identified in this work.

To enhance clarity and support broader dissemination of the results, the authors incorporated an explanatory component on the EPAs into the third round of the Delphi method, alongside the validation stage [17,18,28]. This process led to organizing and condensing the 14 validated EPAs into 7 final EPAs, without compromising the overall findings. All 7 final EPAs exhibited the same validity index and a statistically significant content confirmation index.

The authors conclude that this pioneering study developed and validated 7 EPAs in Pediatric Surgery for the practice of General Pediatricians in Brazil, thereby filling a gap in medical education. The implementation of these EPAs holds the potential to standardize and enhance the training of pediatric residents, significantly contributing to both general pediatric and surgical care in the country.

## Funding

There weren’t any financial funds used in this study.

## Conflicts of interest

The authors declare no conflicts of interest.

## References

[1] Iobst W.F., Sherbino J., ten Cate O., Richardson D.L., Dath D., Swing S.R. (2010). Competency-based medical education in postgraduate medical education. Med Teach.

[2] Frank J.R., Snell L.S., ten Cate O., Holmbo E.S., Carracio C., Swing S.R. (2010). Competency-based medical education: theory to practice. MedTeach.

[3] Caretta-Weyer H.A., Smirnova A., Barone M.A., Frank J.R., Hernandez-Boussard T., Levinson D. (2024). The next era of assessment: building a trustworthy assessment system. Perspect Med Educ.

[4] ten Cate O. (2005). Entrustability of professional activities and competency-based training. Med Educ.

[5] Ten Cate O. (2013). Nuts and bolts of entrustable professional activities. J Grad Med Educ.

[6] Choe J.H., Knight C.L., Stiling R., Corning K., Lock K., Steinberg K.P. (2016). Shortening the miles to the milestones: connecting EPA-based evaluations to ACGME milestone reports for internal medicine residency programs. Acad Med.

[7] ten Cate O., Schumacher D.J. (2022). Entrustable professional activities versus competencies and skills: exploring why different concepts are often conflated. Adv Health Sci Educ Theory Pract.

[8] Peters H., Holzhausen Y., Boscardin C., ten Cate O., Chen H.C. (2017). Twelve tips for the implementation of EPAs for assessment and entrustment decisions. Med Teach.

[9] Carraccio C., Englander R., Gilhooly J., Mink R., Hofkosh D., Barone M.A. (2017). Building a framework of entrustable professional activities, supported by competencies and milestones, to bridge the educational continuum. Acad Med.

[10] Duarte B.S., Vasconcelos M.V., Peixoto A.V. (2021). Clinical skills assessment and feedback in pediatric residency. Rev Bras Educ Med.

[11] O’Keeffe M. (2014). Clinical competence in developmental-behavioural paediatrics: Raising the bar. J Paediatr Child Health.

[12] Larrabee J.G., Agrawal D., Trimm F., Ottolini M. (2020). Entrustable professional activities: correlation of entrustment assessments of pediatric residents with concurrent subcompetency milestones ratings. J Grad Med Educ.

[13] Mink R.B., Schwartz A., Herman B.E., Turner D.A., Curran M.L., Myers A. (2018). Validity of level of supervision scales for assessing pediatric fellows on the common pediatric subspecialty entrustable professional activities. Acad Med.

[14] Kerth J.L., van Treel L., Bosse H.M. (2022). The use of entrustable professional activities in pediatric postgraduate medical education: a systematic review. Acad Pediatr.

[15] Romão G.S. (2023). UNIVERSIDADE federal do MARANHÃO. diretoria de tecnologias na EDUCAÇÃO. curso de formação de preceptores da educação em saúde - FORPRES - módulo 1: o eu preceptor no contexto da educação em saúde.

[16] Romão G.S. (2022). The role of entrustable professionals activities in the training of specialists in gynecology and obstetrics. Rev Bras Ginecol Obstet.

[17] Costa M.L., Rego M.A., Rodrigues F.C., Pinheiro S.S., Deus M.O., Moura A.S. (2024). Validation of entrustable professional activities for use in neonatal care residency programs. J Pediatr (Rio J).

[18] RESOLUÇÃO N° 1, DE 29 DE DEZEMBRO DE 2016 da COMISSÃO NACIONAL DE RESIDÊNCIA MÉDICA publicada no Diário Oficial da União no dia 30 de dezembro de 2016 nas páginas 200 e 201.

[19] Englander R., Carraccio C. (2014). From theory to practice: making entrustable professional activities come to life in the context of milestones. Acad Med.

[20] Schumacher D.J., West D.C., Schwartz A., Li S.T., Millstein L., Griego E.C. (2020). Longitudinal assessment of resident performance using entrustable professional activities. JAMA Network Open.

[21] Anderson M., Burke A., Calaman S., Kuo A., Larrabee J. (2025).

[22] Czaja A.S., Mink R.B., Herman B.E., Weiss P., Turner D.A., Curran M.L. (2024). Exploring factors for implementation of EPAs in pediatric subspecialty fellowships: a qualitative study of program directors. J Med Educ Curric Dev.

[23] Amare E.M., Siebeck M., Sendekie T.Y., Fischer M.R., Berndt M. (2022). Development of an entrustable professional activities (EPA) framework to inform surgical residency training programs in Ethiopia: a three-round national delphi method study. J Surg Educ.

[24] ten Cate O., Nel D., Hennus M.P., Peters S., Romão G.S. (2024). For which entrustable professional activities must medical students be prepared if unsupervised patient care without further training is an expectation? An international Global South study. BMJ Glob Health.

[25] Hennus M.P., Nusmeier A., van Heesch G.G., Riedijk M.A., Schoenmaker N.J., Soeteman M. (2021). Development of entrustable professional activities for paediatric intensive care fellows: a national modified Delphi study. PLoS One.

[26] Diamond I.R., Grant R.C., Feldman B.M., Pencharz P.B., Ling S.C., Moore A.M. (2014). Defining consensus: a systematic review recommends methodologic criteria for reporting of Delphi studies. J Clin Epidemiol.

[27] Villiers M.R., Villier P.J.T., Kent A.T. (2005). The Delphi technique in health sciences education research. Med Teach.

[28] Iqbal M.Z., Könings K.D., Al-Eraky M.M., van Merriënboerb J.J.G. (2021). Entrustable professional activities for small-group facilitation: a validation study using modified Delphi Technique. Teach Learn Med.

[29] de Jesus L.E., Rosina A.G., Ranha B.C., Campos M.C. (2023). Pediatric surgery in Brazil: bittersweet. J Pediatric Surgery Open.

[30] Bustorff-Silva J., Miranda M.L., Rosendo A., Gerk A., Oliveira-Filho A.G (2023). Evaluation of the regional distribution of the pediatric surgery workforce and surgical load in Brazil. World J Pediatr Surg.

